# Histamine H_3_ Receptor Signaling Regulates the NLRP3 Inflammasome Activation in C2C12 Myocyte During Myogenic Differentiation

**DOI:** 10.3389/fphar.2021.599393

**Published:** 2021-05-31

**Authors:** Yan Chen, Yuan Ma, Jin Jin Feng, Yi He Wang, Tian Fang Li, Katariina Nurmi, Kari K. Eklund, Jian Guo Wen

**Affiliations:** ^1^ Urodynamic Center and Department of Urology, Institute of Clinical Medicine, The First Affiliated Hospital of Zhengzhou University, Zhengzhou, China; ^2^ Helsinki Rheumatic Diseases and Inflammation Research Group, Clinicum Helsinki University, Helsinki, Finland; ^3^ Translational Immunology Research Program, University of Helsinki, Helsinki University Clinicum, Helsinki, Finland; ^4^ Department of Rheumatology, The First Affiliated Hospital of Zhengzhou University, Zhengzhou, China; ^5^ Department of Medicine, Division of Rheumatology, Helsinki University Central Hospital, and Orton Orthopedic Hospital, Helsinki, Finland

**Keywords:** histamine H_3_ receptor, myogenesis, inflammation, NLRP3 inflammasome, IL-1β, TNFα, C2C12 myocyte

## Abstract

NLRP3 inflammasome has been implicated in impaired post-injury muscle healing and in muscle atrophy. Histamine receptors play an important role in inflammation, but the role of histamine H_3_ receptor (H_3_R) in myocyte regeneration and in the regulation of NLRP3 inflammasome is not known. We studied the effects of H_3_R signaling on C2C12 myocyte viability, apoptosis, and tumor necrosis factor alpha (TNFα)-induced NLRP3 inflammasome activation during striated myogenic differentiation at three time points (days 0, 3, and 6). Expression of *Nlrp3*, interleukin-1β (IL-1β), and myogenesis markers were determined. TNFα reduced overall viability of C2C12 cells, and exposure to TNFα induced apoptosis of cells at D6. Activation of H_3_R had no effect on viability or apoptosis, whereas inhibition of H_3_R increased TNFα-induced apoptosis. Stimulation of C2C12 cells with TNFα increased *Nlrp3* mRNA expression at D3 and D6. Moreover, TNFα reduced the expression of myogenesis markers MyoD1, Myogenin, and Myosin-2 at D3 and D6. H_3_R attenuated TNFα-induced expression of *Nlrp3* and further inhibited the myogenesis marker expression; while H_3_R -blockage enhanced the proinflammatory effects of TNFα and increased the myogenesis marker expression. TNFα-induced secretion of mature IL-1β was dependent on the activation of the NLRP3 inflammasome, as shown by the reduced secretion of mature IL-1β upon treatment of the cells with the small molecule inhibitor of the NLRP3 inflammasome (MCC950). The activation of H_3_R reduced TNFα-induced IL-1β secretion, while the H_3_R blockage had an opposite effect. In conclusion, the modulation of H_3_R activity regulates the effects of TNFα on C2C12 myocyte differentiation and TNFα-induced activation of NLRP3 inflammasome. Thus, H_3_R signaling may represent a novel target for limiting postinjury muscle inflammation and muscle atrophy.

## Introduction

Inflammation can complicate post-injury muscle healing and slow down the regenerative repair process (Hofer et al., 2014). However, the factors underlying the post injury inflammation are poorly known. It is believed that activation of the inflammasomes, with ensuing production of highly proinflammatory cytokines, IL-1β and IL-18, may play a key role by initiating the inflammatory reaction (Mangan et al., 2018). Nucleotide-binding domain and leucine-rich repeat containing family (NLR), pyrin domain containing 3 (NLRP3) inflammasome is the most widely studied inflammasome. It is activated by a myriad of factors, including exogenous pathogens and endogenous danger signals, and has been implicated in a variety of immunological (Mulay et al., 2018) and non-immunological diseases (Hughes et al., 2016). The NLRP3 inflammasome is a multi-protein complex, consisting of the sensor protein NLRP3, apoptosis-associated speck-like protein containing a CARD (ASC), and the precursor of caspase 1 (pro-caspase-1). Upon activation, NLRP3 is associated with pro-caspase-1 via the adaptor protein ASC resulting in autoactivation of pro-caspase-1 into its active form. The active caspase-1 then catalyzes the cleavage of the inactive precursors of interleukin 1β (pro-IL-1β) and interleukin 18 (pro-IL-18) into their active secreted forms, thus triggering the inflammatory storm (Man and Kanneganti, 2015). NLRP3 inflammasome is mainly expressed in innate immune cells such as neutrophils, macrophages, monocytes, and dendritic cells, but low expression levels are seen in other cell types and tissues (Latz et al., 2013; Elliott and Sutterwala, 2015; Guo et al., 2015; Jo et al., 2016). NLRP3 has been shown to be expressed also in myocytes (Wei et al., 2020) and it has been implicated in the pathogenesis of inflammatory myopathies (Boursereau et al., 2018).

After exercise, skeletal muscle can secrete many bioactive molecules, known as myokines, and tumor necrosis factor alpha (TNFα) is one of the most common exercise-regulated myokines (also named adipo-myokines) (Görgens et al., 2015), which can induce autophagy and apoptosis in mouse C2C12 myoblasts and myotubes (Gallo et al., 2015). The inflammation-associated insulin resistance of C2C12 myoblasts and myotubes has been suggested to be mediated by the activation of NLRP3 inflammasome (Cho and Kang, 2015).

There are four known histamine receptors, of which histamine H_3_ receptor (H_3_R) is mainly expressed in immune and nerve cells, and is involved in nerve conduction, muscle contraction, gastrointestinal neuromodulation, and inflammation (Branco et al., 2018). We found previously that H_3_R is expressed in C2C12 myoblasts and in primary adult mid-urethral striated muscles (Chen et al., 2015; Chen et al., 2015). In C2C12 myoblasts the expression of H_3_R was increased during their myogenic differentiation into striated myocytes, and in myocytes the activation of the H_3_R facilitated the relaxation of the cells by limiting the cytoplasmic calcium peak (Chen et al., 2015). Histamine and histamine receptors possess immunomodulatory effects (Branco et al., 2018). Histamine has been reported to dampen the inflammatory effects of lipopolysaccharides, and in particular, histamine has been shown to inhibit the secretion of IL-1β in microglial cells (Barata-Antunes et al., 2017). In present study we explore further the role of H_3_R in regulation of myoblast differentiation and myocyte function. We hypothesized that H_3_R could modulate the secretion of IL-1β by regulating the activation of the NLRP3 inflammasome. By utilizing highly selective H_3_R agonists (methimepip, Met) (Chen et al., 2015; Panula et al., 2015), and H_3_R blocker (ciproxifan, CPX) (Chen et al., 2015; Panula et al., 2015) we show that activation of H_3_R signaling mitigates the TNFα-induced NLRP3 inflammasome activation, and thus H_3_R signaling could represent a potential target for treatment in conditions involving muscle inflammation and inflammation induced muscle atrophy.

## Experimental

### Materials

The cell culture medium, Dulbecco’s modified Eagle’s medium (DMEM), was purchased from Biowhittaker/Lonza, penicillin-streptomycin from Cambrex/Lonza, Glutamine [Gibco^®^ GlutaMAX™ Supplement (35050061)], and heat inactivated fetal bovine serum (FBS, 10100147) was from Thermo Fisher Scientific. The primers were designed and purchased from Sangon Biotech, China. Mouse IL-1β enzyme-linked immunosorbent assay (ELISA) Kit (D720335-0096) and recombinant murine TNFα (C600052-0005) were purchased from Sangon Biotech, China. The Cell Counting Kit (CCK-8) (C0038) and Annexin V-FITC/propidium iodide (PI) Apoptosis Detection Kit (C1063) were purchased from Beyotime Corporation, China. H_3_R agonist methimepip (Met) (sc-204080) was from Santa Cruz Corporation, and ciproxifan (CPX) (C6492), H_3_R blocker, was from SIGMA Inc. MCC950 (CP-456773, 210826-40-7, AbMole, China) was purchased from AbMole Inc.

### Cell Culture

Mouse C2C12 myoblasts were obtained from American Type Culture Collection, and maintained in DMEM supplemented with 10% fetal bovine serum (FBS), 1% antibiotics (100 U/ml penicillin and 100 μg/ml streptomycin (GIBCO #15140-122), and glutamine at 37°C in humidified 5% CO_2_-in-air. In the differentiation medium, FBS was reduced from 10 to 1%, but otherwise the differentiation medium had the same composition as the growth medium. The cells were passaged at 80% confluence by trypsinization (0.5% trypsin in 0.5 mM EDTA, Gibco BRL, Life Technologies, Gaithersburg, MD).

### C2C12 Cell Stimulation

C2C12 cells were seeded in 96-well plates (1.6 × 10^4^ cells/well), 12-well plates (5 × 10^4^ cells/well), or six-well plates (4 × 10^5^/well) in the growth media and cultured for three days before changing to differentiation media. The differentiation time points are calculated as follows: D0 represent the cells that were collected 3 days after seeding and have not received differentiation media; D3 cells received differentiation media 3 days after seeding, and were cultured for 3 days in differentiation media; D6 received differentiation media 6 days after seeding, and were cultured for 6 days in differentiation media. Collected samples were subjected on analysis of proliferation and viability (CKK-8 assay), apoptosis and necrosis (AnnexinV/PI assay) and gene expression (qPCR). The experimental protocol is described as a flowchart in Supplementary Figure S1A.

Experimental groups 1–4 represent the non-differentiated myocytes, that were collected on differentiation day 0 (D0) 12 h after stimulations with TNFα, TNFα+ Met, TNFα+ CPX, or left unstimulated.

Groups 5–8 were cultured for 3 days (D3) in differentiation media, after which they were stimulated for 12 h with TNFα, TNFα+ Met, TNFα+ CPX, or left unstimulated (Differentiation media was changed on D1 and D2).

Groups 9–12 represent the fully differentiated myocytes that were cultured for 6 days in differentiation media. D6 cells were collected after 12 h stimulations with TNFα, TNFα+ Met, TNFα+ CPX, or left unstimulated (Differentiation media was changed on D3, D4, and D5).

All the above-mentioned treatments were applied on cells with identical treatments with or without 1 h preincubation of the cells in the presence of MCC950 (1 µM), which was left on the cells during stimulations. Experiment media were collected for ELISA, and cell lysates were subjected to western blot analysis (Supplementary Figure S1B). Simultaneously cultured cells with Met or CPX alone were analyzed for apoptosis by flow cytometry (FCM).

### Cell Count Kit-8 (CCK-8) Assay

The cell count kit-8 (CCK-8), a rapid and highly sensitive detection kit for cell proliferation and cytotoxicity, was used to analyze the cell viability according to the manufacturer’s protocol (Beyotime Corporation, Shanghai, China).

### Apoptosis Detection

Cell apoptosis was detected with FCM. AnnexinV/PI apoptosis detection kit was used according to the manufacturer’s protocols (Beyotime Corporation, Shanghai, China). The method is based on the cytofluorimetric assay established by Vermes et al. The kit discriminates intact cells (Annexin-/PI-) from apoptotic (Annexin +/PI−) and necrotic cells (Annexin +/PI+) (Vermes et al., 1995).

### Quantitative Real-Time RT-PCR Analysis

C2C12 cells were harvested and their total cellular RNA was purified using RNeasy Mini Kit (QIAGEN, Düsseldorf, Germany) according to the manufacturer’s instructions. Briefly, 1 μg of total RNA was reverse transcribed using iScript cDNA Synthesis Kit (BioRad Laboratories, Hercules, CA). Quantitative RT-PCR (qPCR) was performed with 100 ng first-strand cDNA using iQ SYBR Green Supermix (Bio-Rad Laboratories, Hercules, CA) in iCycler iQ5 Multicolor Real-Time PCR Detection System (Bio-Rad Laboratories, Hercules, CA). The copy numbers of mRNA in the samples analyzed were determined in triplicate and normalized against PBGD gene as an endogenous control and the relative units were calculated using the comparative Ct method. The following primers were designed with the NCBI Primer Blast program:

*Il1b* sense primer (F, forward): 5′-GCA​ACT​GTT​CCT​GAA​CTC​AAC​T-3′, anti-sense primer (R, reverse): 5′-ATC​TTT​TGG​GGT​CCG​TCA​ACT-3′; *Nlrp3* sense primer (F, forward): 5′-ATT​ACC​CGC​CCG​AGA​AAG​G-3′, anti-sense primer (R, reverse): 5′-TCG​CAG​CAA​AGA​TCC​ACA​CAG-3′; *MyoD1* sense primer (F, forward): 5′-CCA​CTC​CGG​GAC​ATA​GAC​TTG-3′, anti-sense primer (R, reverse): 5′-AAA​AGC​GCA​GGT​CTG​GTG​AG-3′; Myogenin sense primer (F, forward): 5′-GAG​ACA​TCC​CCC​TAT​TTC​TAC​CA-3′, anti-sense primer (R, reverse): 5′-GCT​CAG​TCC​GCT​CAT​AGC​C-3′; *Myosin-2* sense primer (F, forward): 5′-GTC​AGC​ACC​ATG​TCT​TAT​GGG-3′, anti-sense primer (R, reverse): 5′-TTT​GCC​AAA​TCG​GGA​AGA​GTT-3′; *PBGD* sense primer (F, forward): 5′-AGG​TCG​GTG​TGA​ACG​GAT​TTG-3′, anti-sense primer (R, reverse): 5′-GGG​GTC​GTT​GAT​GGC​AAC​A-3′.

### Western Blotting

Cells were lyzed in 150 µl of Mammal Cell Protein Extraction Reagent (CWBiotech, Beijing, China). The total protein concentration was determined with a Pierce BCA protein assay kit (Trans, Beijing, China). For each sample 40 µg of protein was loaded and separated by sodium dodecyl sulfate-polyacrylamide gel electrophoresis consisting of 5% stacking gel and 12% resolving gel, and the proteins were electrophoretically transferred to a polyvinylidene fluoride (PVDF) membrane. The membranes were blocked with 5% nonfat milk in Tris-buffered saline (pH 7.4) containing 0.1% Tween-20 (TBST). The membranes were incubated overnight at 4 °C with mouse monoclonal antibodies against IL-1β (3A6, mouse mAb #12242S, Cell Signaling Technology, Shanghai, China) in TBST with 5% nonfat milk. The secondary antibody goat anti-mouse IgG, HRP conjugated (CW0102S, CWBiotech, Beijing, China) was used to incubate the membrane for 2 h at room temperature (RT). Labeling was performed using an enhanced chemiluminescence (ECL) system (Thermo Fisher Scientific, Thermo, United States). The density and area of the bands was quantitated.

### ELISA Analyses

C2C12 cells were incubated in the presence of TNFα, with or without Met or TNFα or MCC950 at day D0, D3, D6 for 24 h. Mouse IL-1β concentrations in the cell culture media were analyzed using a commercial enzyme-linked immunosorbent assay (ELISA) kit according to the manufacturer’s protocols (Sangon Biotech, Shanghai, China).

### Statistical Analysis

Calculations were performed using GraphPad InStat3 for Macintosh software. Overall significance level between the stimulated and control groups or between stimulated and inhibited groups was analyzed using one-way ANOVA, with the Dunnett and Tukey-Kramer multiple comparisons tests when appropriate. Statistical significance was set at *p* < 0.05. The data are calculated as mean ± SEM of 12 samples (three repeated experiments, each treatment performed in four replicates).

## Results

### Effects of TNFa, and Modulation of H_3_R Signaling on Myocyte Viability

**FIGURE 1:**
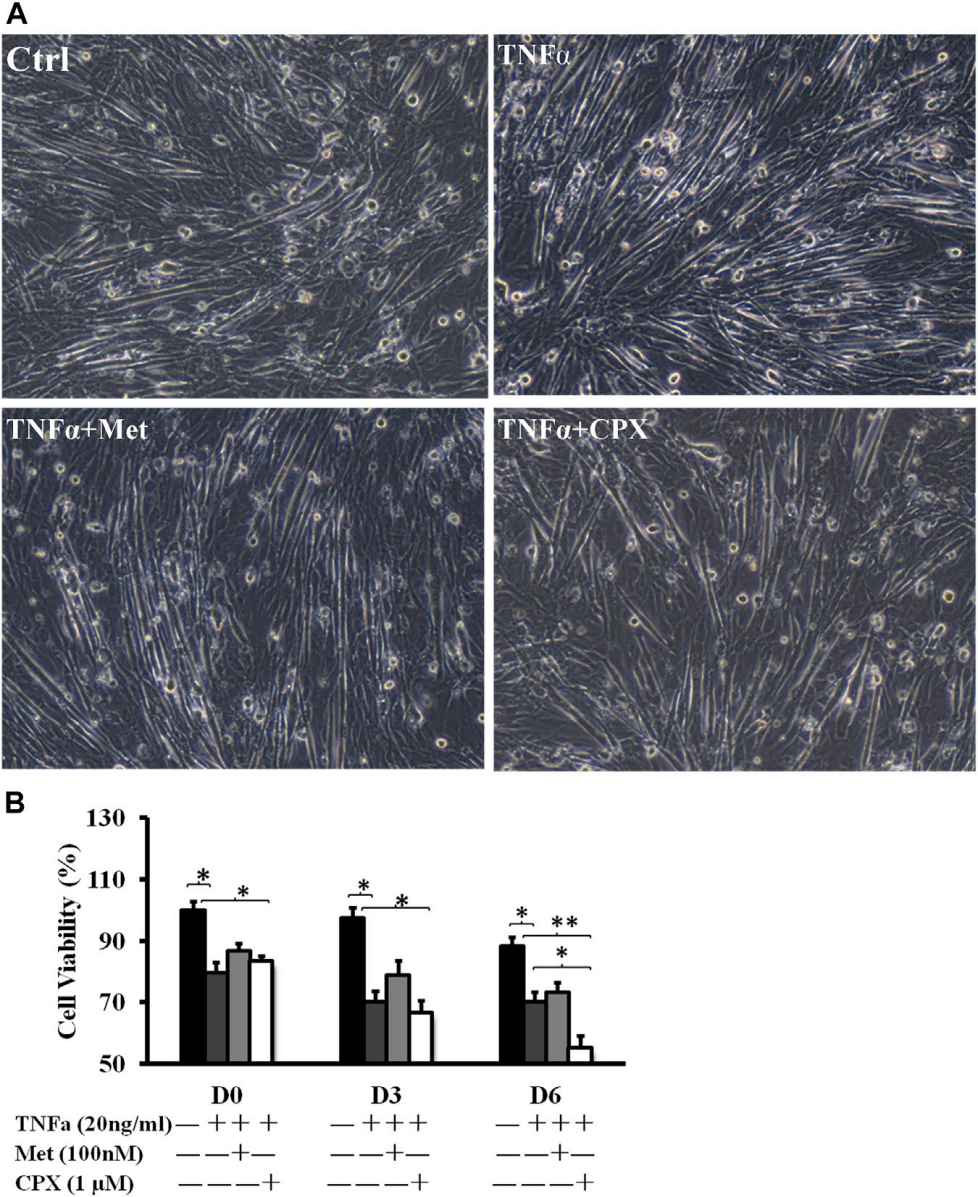
The effect of TNFα, Met and CXP on cell viability. **(A)**: D6 C2C12 cells incubated in the presence of TNFa (20 ng/ml), TNFa + Met (100 nM) or TNFa + CPX (1 µM) for 12 h and observed by phase contrast microscopy (100×). **(B)**: Viability at D0, D3, and D6. C2C12 cells were incubated for 12 h in the presence of TNFa, TNFa + Met or TNFa + CPX. The cell death was detected by cell count kit-8 assay; *p < 0.05, **p < 0.01 vs. control of the same differentiation day (black columns); normalizing D0 ctrl = 100%. There was no statistical difference when TNFa + Met or TNFa + CPX was compared with TNFa-treatment of 0- and 3-differentiation day (dark gray columns). Ctrl, control; TNFa, murine Tumor Necrosis Factor-alpha; Met, methimepip; CPX, ciproxifan. D0, undifferentiated cells; D3, cells differentiated for 3 days; D6, cells differentiated for 6 days.

The C2C12 cells were fully differentiated at D6 in each stimulation (Figure 1A). During C2C12 myogenesis, TNFα decreased cell viability at all the measured time points, days 0, 3, 6 (D0, D3, D6), by 20.5% (*p* < 0.05), 29.8% (*p* < 0.05), and 29.7% (*p* < 0.05), respectively, as compared to the controls. Addition of H_3_R antagonist ciproxifan (CPX) together with TNFα had no additional effect on cell viability at the time points D0 and D3, but further reduced the viability at time point D6 (40.3%, *p* < 0.05). Meanwhile, addition of H_3_R agonist methimepip (Met) together with TNFα had no additional effect on cell viability at any of the time points of D0, D3, and D6 (Figure 1).

### Inhibition of H_3_R Signaling Potentiates TNFα-Induced Apoptosis

**FIGURE 2:**
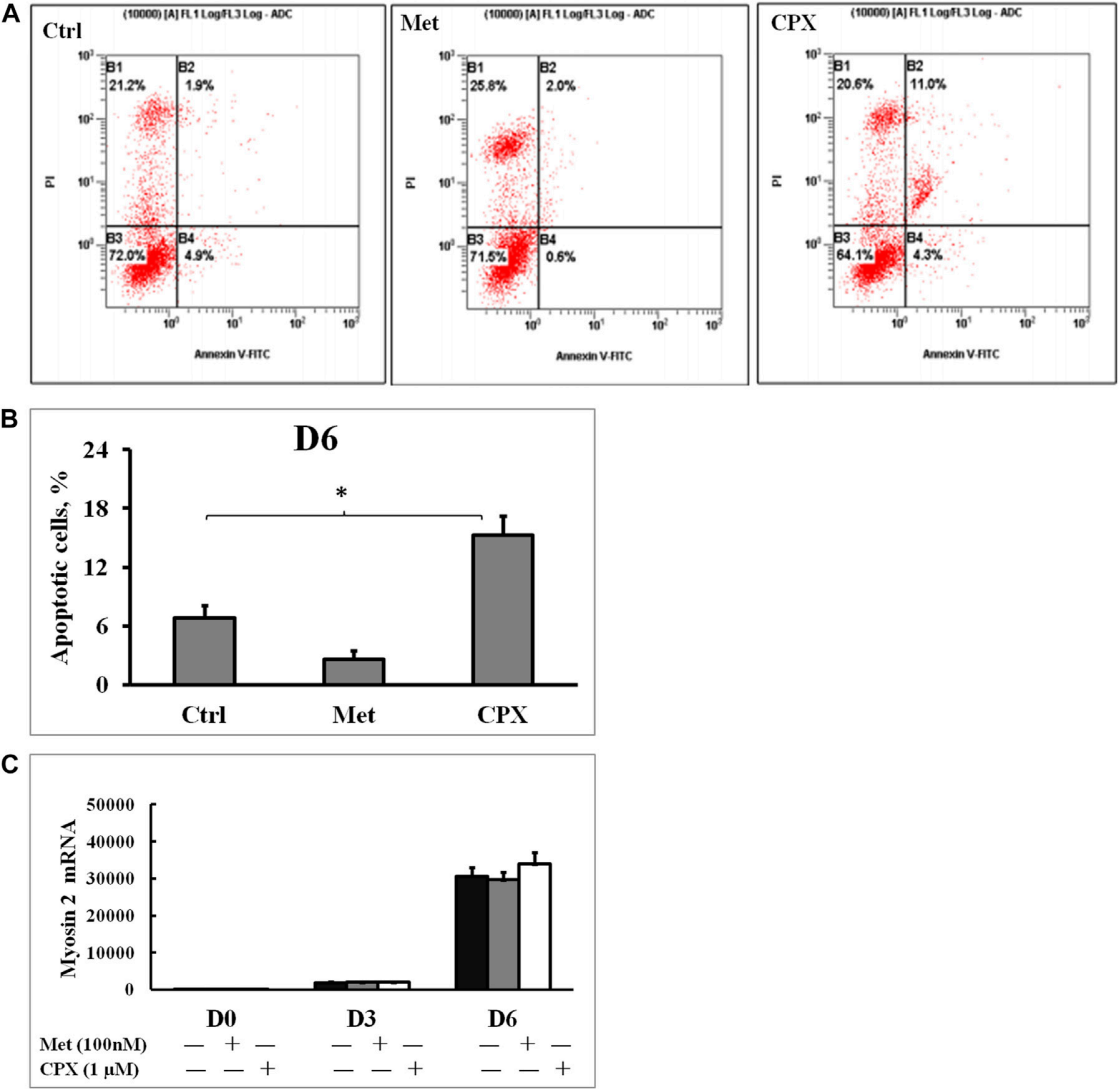
The effect of histamine H3 receptor modulators on cell apoptosis and myogenesis. **(A)**: Apoptotic cells were detected by flow cytometry using Annexin V and propidium iodide staining; percentage of mechanically damaged cells **(top left quadrant)**, live cells **(bottom left)**, early apoptotic cells **(bottom right)**, late apoptotic cells **(top right)**. **(B)**: Percentage of apoptotic cells was calculated by adding the early and the late apoptotic cells for each experimental condition.*p < 0.05 vs. control. **(C)**: The C2C12 myocytes were incubated in the presence of Met (100 nM) or CPX (1 μM) for 12 h at D6, and the effect of H3 receptor inhibition or activation on myogenesis was assessed by analyzing the expression of the late myogenesis marker, myosin 2, mRNA by real time RT‐PCR.*p < 0.05 vs. control of the same differentiation day (black columns); normalizing D0 ctrl=1. There was no statistical difference when Met or CPX was compared with control group (dark gray columns). Ctrl, control; Met, methimepip; CPX, ciproxifan. D0, undifferentiated cells; D3, cells differentiated for 3 days; D6, cells differentiated for 6 days.

**FIGURE 3:**
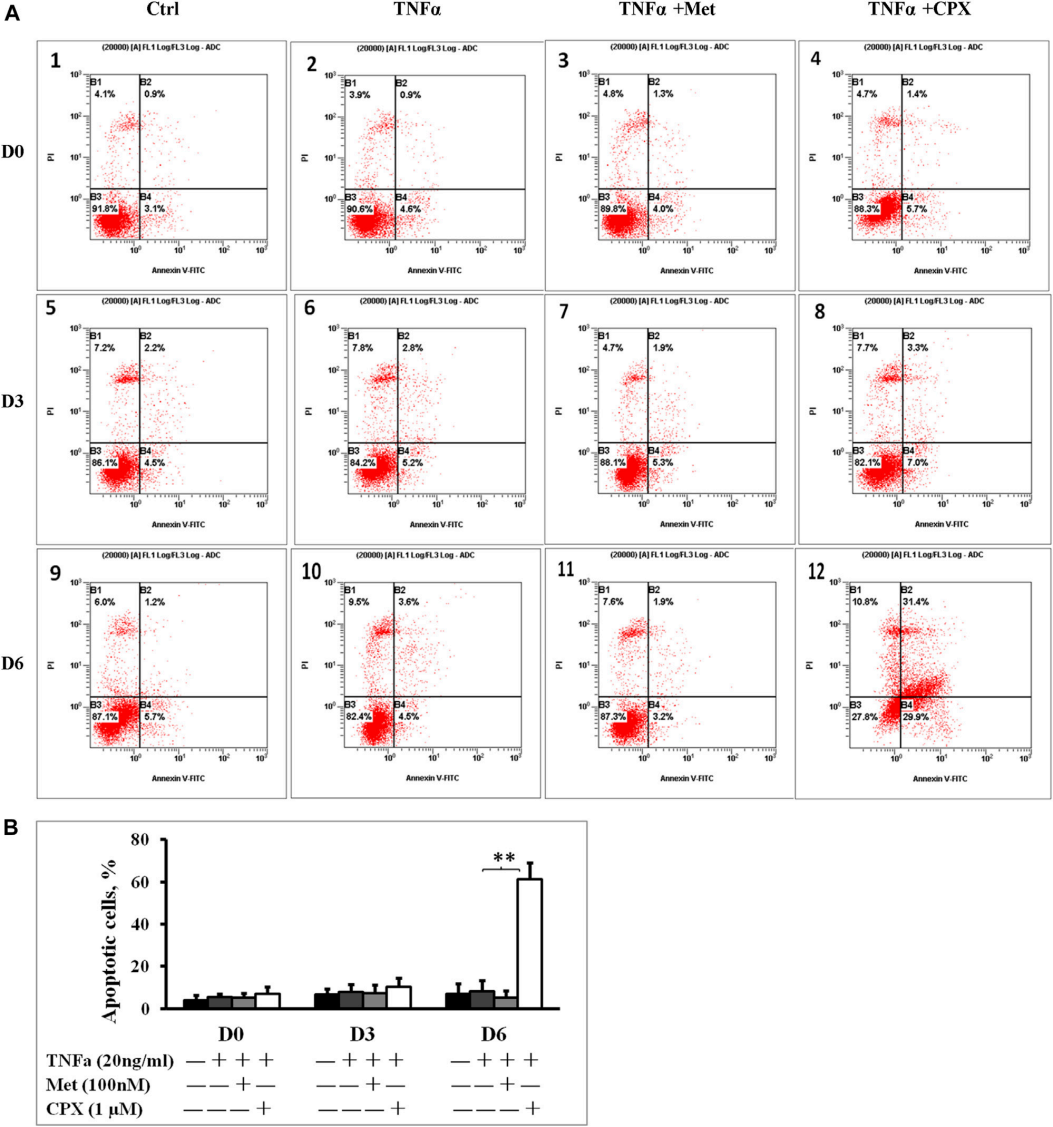
Inhibition of histamine H_3_ receptor enhances apoptosis. **(A)**: C2C12 cells were incubated in the presence of TNFa (20 ng/ml), TNFa + Met (100 nM) or TNFa + CPX (1 µM) at D0, D3, or D6. Apoptotic cells were detected by Annexin V and propidium iodide staining and analyzed by flow cytometry: percentage of cells mechanically damaged **(top left quadrant)**, live cells **(bottom left)**, early apoptotic cells **(bottom right)**, late apoptotic cells **(top right)**. **(B)**: Percentage of apoptotic cells was calculated by adding the early and the late apoptotic cells for each experimental condition. **p < 0.01 vs. TNFα of the same differentiation day (dark gray columns). Ctrl, control; TNFa, murine Tumor Necrosis Factor-alpha; Met, methimepip; CPX, ciproxifan. D0, undifferentiated cells; D3, cells differentiated for 3 days; D6, cells differentiated for 6 days.

Met alone did not induce apoptosis, however, 15.3% of the cells stimulated with CPX were apoptotic (*p* < 0.05) at D6 (Figures 2A,B). Next, we studied the effect of H_3_R inhibition on TNFα-induced apoptosis. During C2C12 myogenesis, apoptosis and necrosis were detected in 4–6.9% of the control cells. Compared to the control cells, TNFα or TNFα +Met did not cause an obvious increase in apoptosis or necrosis during myogenic differentiation (Figure 3). However, treatment with combination of TNFα +CXP induced overall 61.3% (*p* < 0.01) apoptotic cells on D6, among which early apoptosis was observed in 29.9% (*T* = 4.2, *p* < 0.05) of the cells and late apoptosis in 31.4% of the cells (*T* = 5.0, *p* < 0.01) (Figure 3).

### Expression of NLRP3 Receptor mRNA and Myogenesis Markers

**FIGURE 4:**
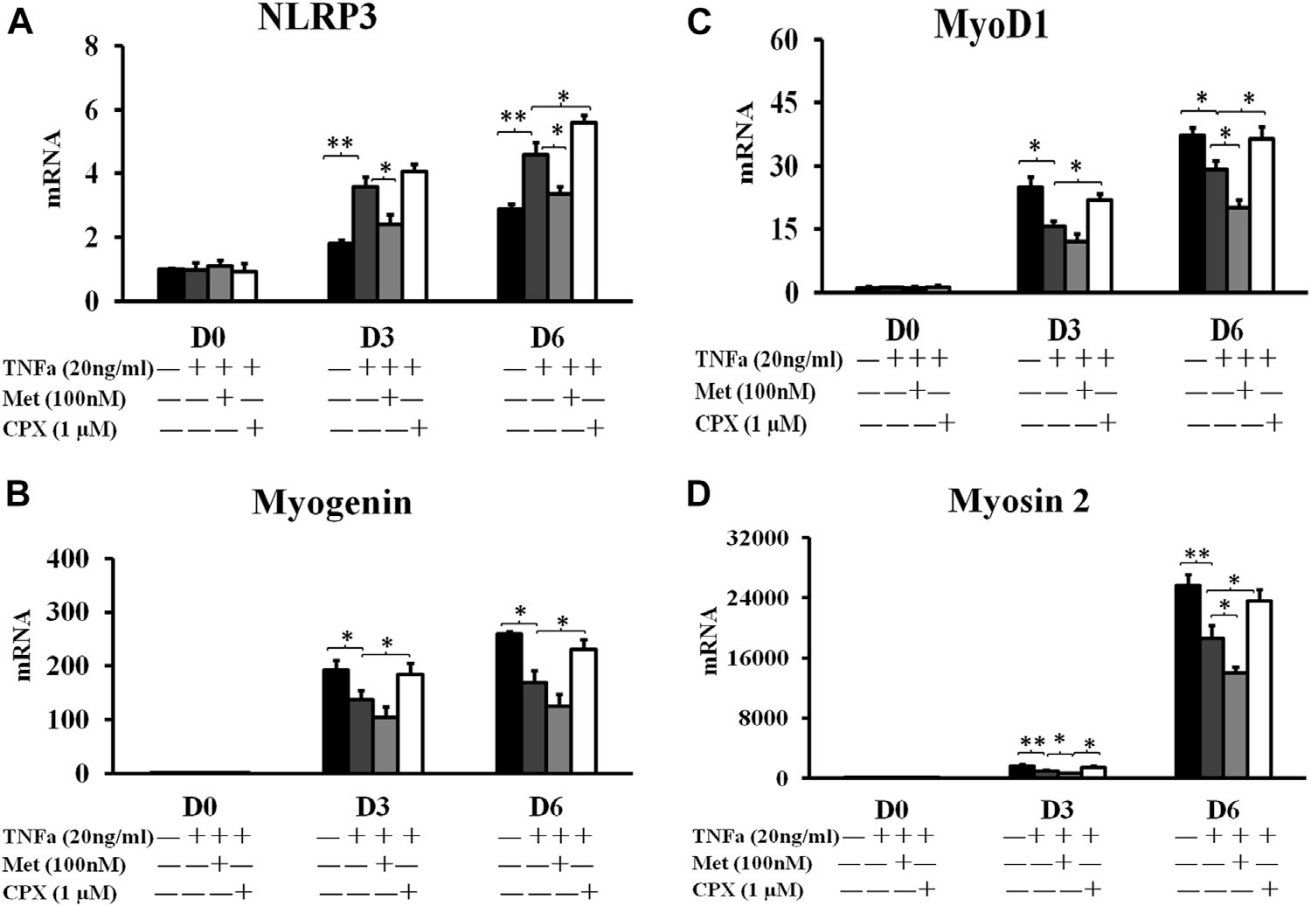
The mRNA expression of inflammasome NLRP3 protein and myogenesis markers. C2C12 cells were incubated in the presence of TNFa (20 ng/ml), TNFa + Met (100 nM) or TNFa + CPX (1 µM) for 12 h at D0, D3, or D6. The mRNA expressions were assessed by real time RT-PCR. *p < 0.05, and **p < 0.01 between two compared groups. Normalizing D0 ctrl = 1 after compared with housekeeping gene PBGD. **(A)**: NLRP3 mRNA expression. **(B)** The mRNA expression of early myogenesis marker, MyoD1. **(C)** The mRNA expression of intermediate myogenesis marker, Myogenin. **(D)** The mRNA expression of late myogenesis marker, myosin 2. Ctrl, control; TNFa, murine Tumor Necrosis Factor-alpha; Met, methimepip; CPX, ciproxifan. D0, undifferentiated cells; D3, cells differentiated for 3 days; D6, cells differentiated for 6 days. NLRP3, Nucleotide-binding domain and leucine-rich repeat containing family, pyrin domain containing three.

As previous study has shown that another proinflammatory cytokine, IL-1β, induces the expression of *Nlrp3* in C2C12 cells (Huang et al., 2017), we studied the involvement of the NLRP3 inflammasome in the TNFα-induced inflammation. We analyzed the *Nlrp3* expression during C2C12 myogenesis on D0, D3 and D6. TNFα induced the expression of *Nlrp3* mRNA up to 201% at D3 (*p* < 0.01) and 190% at D6 (*p* < 0.01) as compared to the controls (Figure 4A). Met reduced the TNFα-induced expression of *Nlrp3* by 33% at D3 (*p* < 0.05) and 30% at D6 (*p* < 0.05), while CPX enhanced the effects of TNFα on *Nlrp3* expression by 24% (*p* < 0.05) at D6 (Figure 4A).

To study the effects of TNFα and H_3_R modulation in myogenic differentiation, we analyzed the expression of the conventional myogenic markers. Myogenic Differentiation 1 (MyoD1) is considered as one of the earliest myogenic markers. MyoD1 is a nuclear protein that belongs to the subfamily of myogenic factors, and it regulates muscle cell differentiation by inducing cell cycle arrest, which is a prerequisite for myogenic initiation (Blais et al., 2005). Myogenin is usually regarded as an early myogenic marker. It is a muscle-specific transcription factor that is involved in coordination of skeletal muscle development or myogenesis and repair, and it is essential for the development of functional skeletal muscle (Blais et al., 2005). Myosin heavy chain 2 (Myosin-2) is a late myogenic marker. It is a member of the class II or conventional myosin heavy chains, and functions in skeletal muscle contraction (Stuart et al., 2016). TNFα reduced the expression of myogenesis markers: MyoD1 at D3 and D6 by 38% (*p* < 0.05) and 22% (*p* < 0.05) (Figure 4C), Myogenin at D3 and D6 by 29% (*p* < 0.05) vs. 35% (*p* < 0.05) (Figure 4B), and Myosin-2 at D3 and D6 by 42% (*p* < 0.01) and 28% (*p* < 0.01) (Figure 4D), respectively. Met enhanced the inhibitory effect of TNFα of the above three markers by 23 and 31% (*p* < 0.05), 22 and 26%, 30% (*p* < 0.05) and 25% (*p* < 0.05) at D3 and D6, respectively, while CPX reduced the inhibitory effect of TNFα on the myogenic marker expression by 40% (*p* < 0.05) and 25%, 35% (*p* < 0.05) and 37% (*p* < 0.05), 50% (*p* < 0.05) and 27% (*p* < 0.05) (Figures 4B–D) respectively. Neither Met nor CPX alone had a clear effect on the mRNA expression of the late myocyte differentiation marker, myosin 2 (*p* > 0.05) (Figure 2C).

### H_3_R Activation Regulates the Levels of Mature IL-1β in TNFα-Treated Cells

**FIGURE 5:**
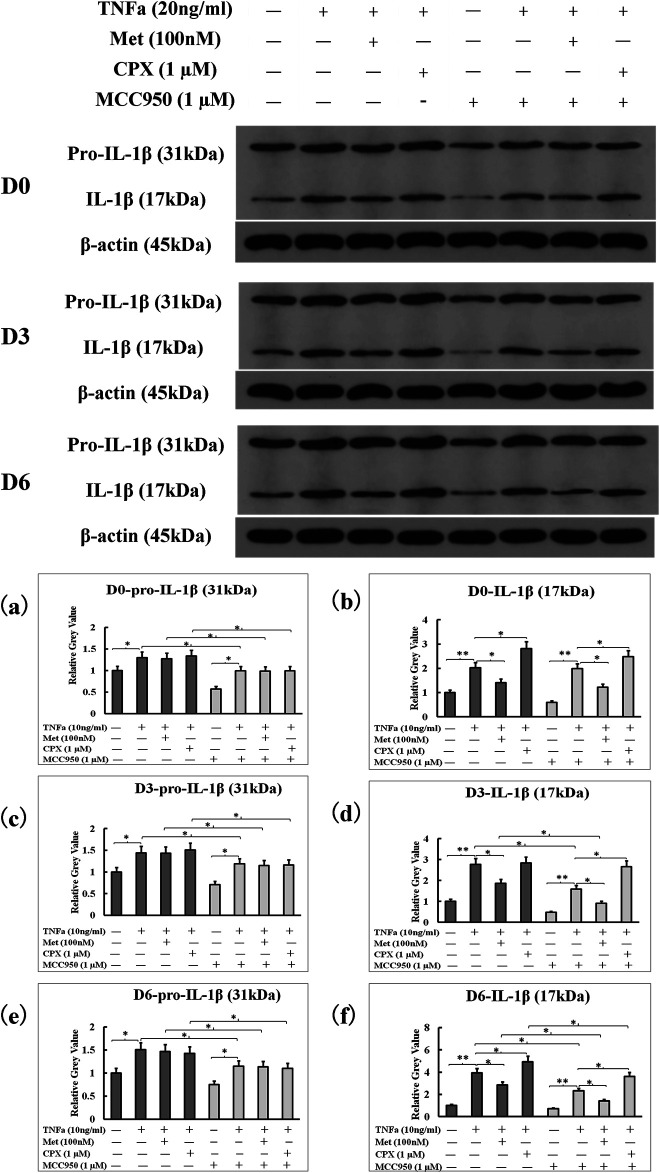
NLRP3 inhibitor, MCC950, inhibited overall pro-IL-1β and IL-1β protein levels induced by TNFα. C2C12 cells were incubated in the presence of TNFa (20 ng/ml), TNFa + Met (100 nM) or TNFa + CPX (1 µM) or overall MCC950 (1 µM) for 12 h at D0, D3, or D6. The pro-IL-1β (31 kDa) and mature IL-1β protein (17 kDa) levels were assessed by westernblot. *p < 0.05, and **p < 0.01 between two compared groups. **(A,B)**: The grey-values of westernblot on pro-IL-1β and IL-1β proteins at D0 were calculated by normalized protein expressions to β-actin. **(C,D)**: The grey-values of westernblot on pro-IL-1β and IL-1β proteins at D3 were calculated by normalized protein expressions to β-actin. **(E,F)**: The grey-values of westernblot on pro-IL-1β and IL-1β proteins at D6 were calculated by normalized protein expressions to β-actin. Ctrl, control; TNFa, murine Tumor Necrosis Factor-alpha; Met, methimepip; pro-IL-1β, the precursor of interleukin 1beta; IL-1β, interleukin 1beta; CPX, ciproxifan. D0, undifferentiated cells; D3, cells differentiated for 3 days; D6, cells differentiated for 6 days.

To study the effects of TNFα and H_3_R modulation on the protein expression IL-1β, we detected the levels of pro-IL-1β (31 kDa) and IL-1β (17 kDa) proteins from cell lysates by western blot. TNFα increased the levels of both the pro-IL-1β (31 kDa) and IL-1β (17 kDa) proteins (*p* < 0.05), and neither Met nor CPX had an effect on TNFα-induced pro-IL-1β expression in D0, D3 and D6 cells (*p* > 0.05). However, Met and CPX modulated the TNFα-induced expression of mature IL-1β protein; Met reduced the level of the mature IL-1β at all the time points (*p* < 0.05) in TNFα-induced D0 and D6 cells, while CPX increased it (*p* < 0.05) at D0, and D6 time points. MCC950, a new specific small molecule NLRP3 inhibitor, reduced the pro-IL-1β (31 kDa) and mature IL-1β (17 kDa) protein levels in each treatment group, except for control group (*p* < 0.05) (Figure 5).

### H_3_R Signaling Modulates the Secretion of IL-1β

**FIGURE 6:**
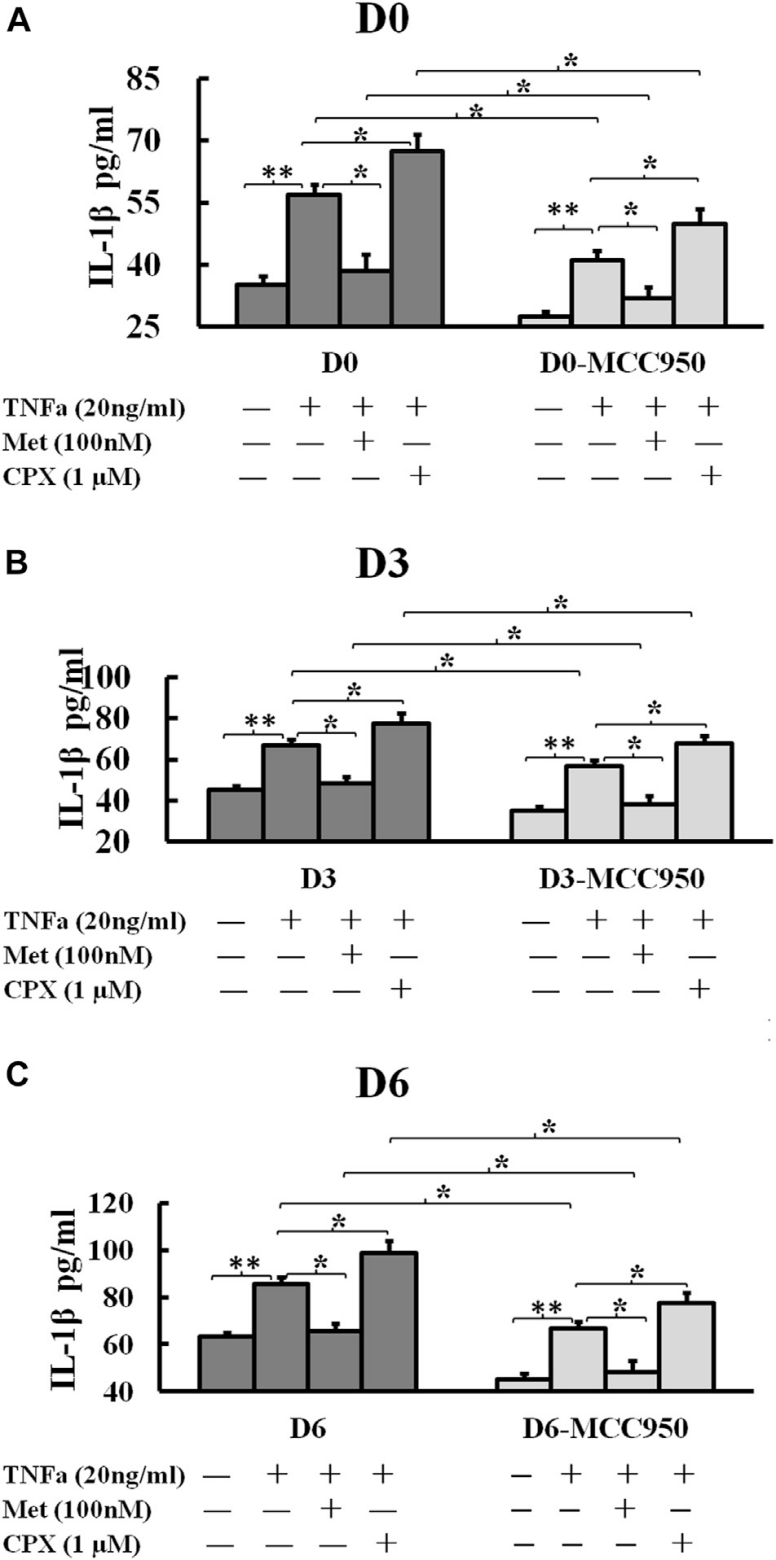
Histamine H_3_ receptor modulates the secretion of IL-1β. C2C12 cells were incubated in the presence of TNFa (20 ng/ml), TNFa + Met (100 nM) or TNFa + CPX (1 µM) or MCC950 (1 µM) for 24 h at D0, D3, or D6. The mature IL-1β protein secretions were assessed by ELISA. *p < 0.05, and **p < 0.01 between two compared groups. **(A)**: IL-1β secretion in D0 cells. **(B)** IL-1β secretion in D3 cells. **(C)** IL-1β secretion in D6 cells. Ctrl, control; TNFα, murine Tumor Necrosis Factor-alpha; Met, methimepip; CPX, ciproxifan; IL-1β, interleukin 1beta.

Since the secretion of mature IL-1β is a marker of inflammasome activation, we next assessed the effect of TNFα and H_3_R modulation on the inflammasome activation by analyzing IL-1β secretion during C2C12 myogenesis. TNFα increased the secretion of IL-1β at D0, D3 and D6 cells to 56.8 (*p* < 0.01), 66.8 (*p* < 0.01), and 85.8 pg/ml (*p* < 0.01), respectively. Met reduced the TNFα-induced secretion of IL-1β to 38.4 (*p* < 0.05), 48.4 (*p* < 0.05), 65.8 (*p* < 0.05) pg/ml, while CPX enhanced the TNFα-induced secretion of IL-1β to 67.5 (*p* < 0.05), 77.5 (*p* < 0.05), 98.9 (*p* < 0.05) pg/ml on D0, D3 and D6 cells, respectively (Figure 6). MCC950 reduced TNFα-induced IL-1β secretion to 41.2 (*p* < 0.05), 56.8 (*p* < 0.05), and 66.8 pg/ml (*p* < 0.05); and MCC950 reduced Met-TNFα-induced IL-1β secretion to 31.9 (*p* < 0.05), 38.4 (*p* < 0.05), 48.4 (*p* < 0.05) pg/ml. MCC950 reduced also CPX-TNFα-induced IL-1β secretion to 49.8 (*p* < 0.05), 67.5 (*p* < 0.05), 77.5 (*p* < 0.05) pg/ml (Figure 6). Thus, our results suggest that H_3_R plays a role in regulation of NLRP3 inflammasome. H_3_R inhibition reduces the TNFα-induced secretion of IL-1β whereas H_3_R activation increases IL-1β secretion in C2C12 myocytes. The secretion of IL-1β was dependent on the NLRP3 inflammasome, as the NLRP3 inhibitor, MCC950, reduced TNFα induced IL-1β secretion in each groups of D0, D3 and D6 cells.

## Discussion

Inflammation is an immunological response to injury and the concomitant release of damage associated molecular patterns (Hofer et al., 2014). The underlying molecular mechanisms are complex but recent studies implicate activation of inflammasomes in sterile inflammatory reactions (Mangan et al., 2018). The present study verified the presence of functional NLRP3-inflammasome in mouse striated C2C12 myocytes (Cho and Kang, 2015; Gallo et al., 2015). TNFα induced both the expressions of pro-IL-1β and also the activation of NLRP3 inflammasome in C2C12 myocytes resulting in secretion of the mature IL-1β protein. TNFα also reduced cell viability and the expression of myogenesis markers, suggesting that it inhibited myocyte differentiation. These findings are in line with previous studies which have shown that IL-1β induces markers of atrophy in C2C12 cells (Huang et al., 2017). Together these findings suggest that TNFα mediated inflammasome activation with ensuing IL-1β secretion could play an important role in postinjury muscle inflammation and inflammation associated muscle atrophy.

Stimulation of H_3_R signaling by Met attenuated the TNFα-induced activation of NLRP3 inflammasome, whereas, inhibition of H_3_R signaling enhanced the inflammasome activation by TNFα. Recently Gao et al. showed that loratadine, a histamine H_1_ receptor blocker, inhibits the activation of NLRP3 inflammasome (Gao and Zhang, 2020), but there are no studies addressing the role of H_3_R in regulation of NLRP3 inflammasome. The effect of TNFα on myogenic differentiation is affected by the conditions used and also on concentration of TNFα. Low levels of TNFα are required for the differentiation, but on the other hand, especially at higher concentration, such as during inflammation, TNFα has been shown to increase myoblast proliferation, and to prevent the cells entering to cell cycle arrest that is a prerequisite for the initiation of myoblast differentiation (Grounds and Torrisi, 2004; Chen et al., 2007; Alter et al., 2008). TNFα reduced the expression of the earliest differentiation markers on the earlier day three time point slightly more than at later sixth day time point. The differences between the time points are probably explained by the number of the cells that have entered the cell cycle arrest before TNFα treatment, because TNFα does not inhibit the differentiation after the cells have entered the cell cycle arrest (Alter et al., 2008), and by day sixmore cells had entered the cell cycle arrest and started differentiation than by day three, and probably therefore the effect of TNFα on differentiation was slightly mitigated.

Activation of H_3_R further slowed myogenic differentiation in the presence of TNFα. The mechanism remains to be elucidated but one possibility is that H_3_R activation directs cell resources into processes of reducing inflammation, thus halting the differentiation. In carcinoma cells H_3_R -mediated activation of protein kinase C α inhibited the growth of cholangiocarcinoma and hepatocellular carcinoma cells (Francis et al., 2009; Yu et al., 2019). The finding that blocking the H_3_R by CPX in the presence of TNFα enhanced cell differentiation, is also in line with studies on cancer cells reporting that inhibition of histamine receptor H_3_ suppresses the growth and metastasis in human non-small cell lung cancer cells (Zhao et al., 2020) thus potentially promoting differentiation. Moreover, the enhanced inflammation induced by CPX + TNFα could also direct cells into apoptosis resulting in the release of ATP, which further activates the NLRP3 inflammasome (Jo et al., 2016).

## Conclusion

TNFα induces NLRP3 inflammasome activation and inhibits myogenesis in striated C2C12 cells during myogenic differentiation. Histamine H_3_R signaling slowed the differentiation and inhibited TNFα induced NLRP3 inflammasome activation. Inhibition of H_3_R signaling enhanced the differentiation and TNFα-induced inflammation. These findings implicate a regulatory role for histamine H_3_R in NLRP3 inflammasome activation. This beneficial effect of H_3_R activation should be balanced with the potentially disadvantageous effect of further slowing down the myocyte differentiation in the presence of TNFα. H_3_R modulation could represent a lucrative target in the treatment of postinjury muscle inflammation and atrophy.

## Data Availability

The original contributions presented in the study are included in the article/Supplementary Material, further inquiries can be directed to the corresponding author.
