# Supplementary material for: Histamine H3 Receptor Signaling Regulates the NLRP3 Inflammasome Activation in C2C12 Myocyte During Myogenic Differentiation
Source: Front Pharmacol. 2021 May 31;12:599393. doi: 10.3389/fphar.2021.599393 (PMC8202077; doi:10.3389/fphar.2021.599393)
Supplement: Supplementary file 1 [file DataSheet1.PDF]

**Supplimental File**

**Figure 1. The flowchart of experimental stimulations process in C2C12 cells' myogenic differentiaon**

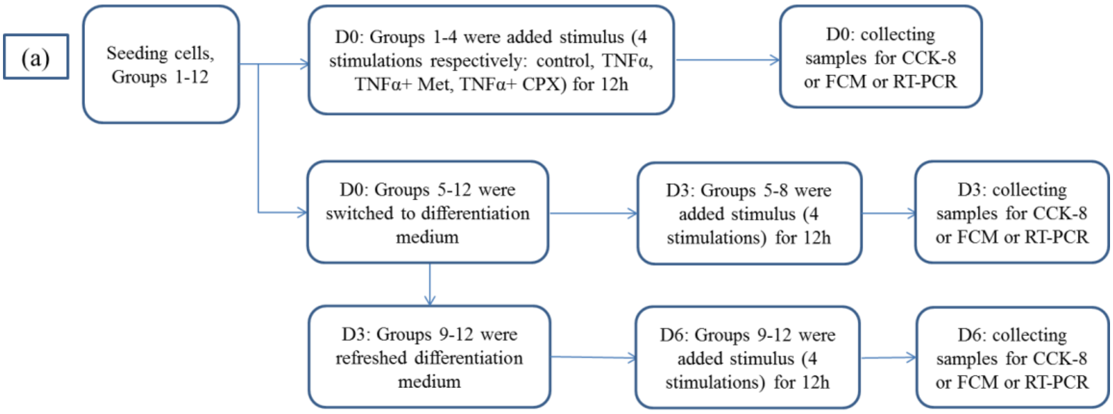


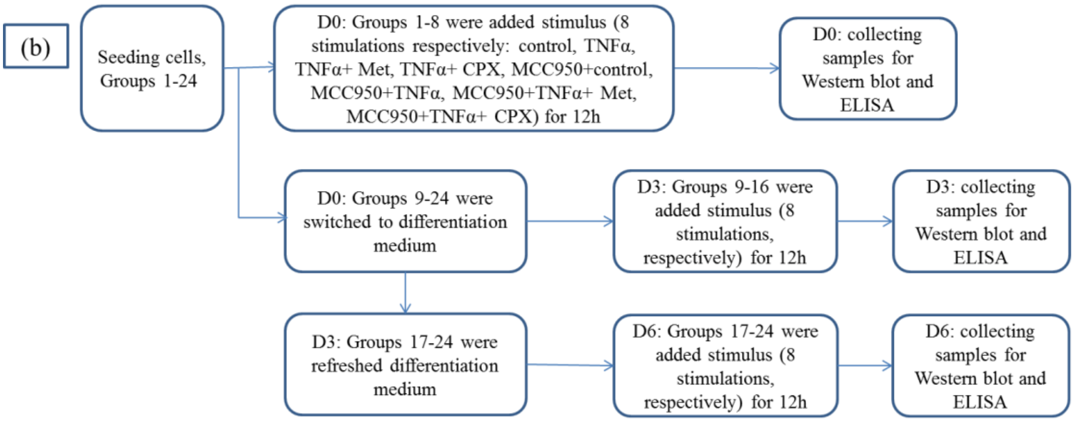


Notes:
TNFa, murine Tumor Necrosis Factor-alpha, 20 ng/ml;
Met, methimepip, 100 nM; CPX, ciproxifan, 1 μM.
MCC950, NLRP3 inhibitor, 1 μM;

D0, undifferentiated cells; D3, cells differentiated for 3 days; D6, cells differentiated for 6 days.

CCK-8, Cell count kit-8 assay; FCM, Flow cytometry AnnexinV/PI apoptosis detection; RT-PCR, Reverse Transcription-Polymerase Chain Reaction;
WB, Western blotting; ELISA, enzyme-linked immunosorbent assay
